# Outcomes and Health Economics of Stroke using Rhythmic Auditory Stimulation (OrcHESTRAS): a protocol for a pragmatic, decentralized, longitudinal, multi-phase, withdrawal with randomized re-treatment trial of MR-001 in chronic stroke

**DOI:** 10.1186/s13063-025-09415-3

**Published:** 2026-01-08

**Authors:** Sabrina R. Taylor, Lou N. Awad, Cecilia A. Carlowicz, Yuri A. Maricich, Seth P. Finklestein, Erika H. Riley, Brian A. Harris, Ryan T. Pohlig, Francois A. Bethoux

**Affiliations:** 1 Department of Clinical Trials and Medical Affairs, MedRhythms, Inc., Portland, ME USA; 2 Department of Physical Therapy, Sargent College of Health and Rehabilitation Sciences, Boston University, Boston, MA USA; 3 Independent Physician-Scientist, Cambridge, MA USA; 4 Stroke Service, Department of Neurology, Massachusetts General Hospital, Boston, MA USA; 5 Department of Medicine, Massachusetts General Hospital, Boston, MA USA; 6 Biostatistics Core Facility, University of Delaware, Newark, DE USA; 7 Department of Physical Medicine and Rehabilitation, Cleveland Clinic Neurological Institute, Cleveland, OH USA

**Keywords:** Stroke rehabilitation, Rhythmic auditory stimulation, Gait, Neuromodulation, Digital therapeutics, Home-based intervention, Mobility, Walking, Neurorehabilitation, Healthcare resource utilization

## Abstract

**Background:**

Persistent gait deficits limiting mobility, independence, and quality of life are common after stroke. These deficits also increase fall risk, hospitalizations, and mortality, driving substantial clinical and economic burden. Interventions that improve gait may reduce these risks and associated costs. Rhythmic auditory stimulation (RAS) is a validated technique that enhances gait parameters, including speed, cadence, and stride symmetry in stroke rehabilitation. MR-001 is an autonomous neurorehabilitation system that delivers personalized RAS for home-based walking rehabilitation. This study incorporates pragmatic and controlled design elements, consistent with real-world implementation settings, to evaluate engagement, clinical effectiveness, durability of response, and health economic impact of MR-001 in people living with gait impairment after stroke.

**Methods:**

This decentralized, longitudinal trial combines a pragmatic single-arm intervention phase with a randomized re-treatment phase. Approximately 225 participants with chronic stroke and gait deficit will be enrolled. In Step 1, all participants receive 12 weeks of MR-001 therapy (30 min, 3 times per week) followed by a 12-week washout. In Step 2, participants are randomized to either 12 additional weeks of MR-001 or a 24-week extended washout. The primary endpoint is user engagement, defined as the proportion achieving at least moderate engagement during Step 1. Secondary endpoints include walking endurance, durability of gains, quality of life, activities of daily living, social isolation, and cognitive function. Exploratory analyses include changes to general mobility, effects of re-treatment, subgroup analyses by baseline function and engagement, proportion and predictors of responders, and healthcare resource utilization assessed through claims data.

**Discussion:**

This pragmatic trial evaluates MR-001, a technology-enabled, autonomous neurorehabilitation system. The design expands on earlier work to assess engagement, durability, and re-treatment while linking clinical outcomes to claims data to generate preliminary evidence on the economic impact of home-based RAS. Findings will inform strategies to optimize adherence, support payer reimbursement, and guide the integration of MR-001 into long-term stroke care.

**Trial registration:**

ClinicalTrials.gov NCT06051539. Registered on 20 September 2023 https://clinicaltrials.gov/study/NCT06051539.

## Administrative information

Note: the numbers in curly brackets in this protocol refer to SPIRIT checklist item numbers. The order of the items has been modified to group similar items (see http://www.equator-network.org/reporting-guidelines/spirit-2013-statement-defining-standard-protocol-items-for-clinical-trials/).

**Table:** 

Title {1}	Outcomes and Health Economics of Stroke using Rhythmic Auditory Stimulation (OrcHESTRAS): a protocol for a pragmatic, decentralized, longitudinal, multi-phase, withdrawal with randomized re-treatment trial of MR-001 in chronic stroke
Trial registration {2a and 2b}	ClinicalTrials.gov, identifier: NCT06051539. Registered on 20 September 2023
Protocol version {3}	Version 4, dated 21 September 2025
Funding {4}	The study is funded by MedRhythms, Inc.
Author details {5a}	Sabrina R. Taylor*, PhD, CCRP (ORCID: 0000-0003-4867-1922) Department of Clinical Trials and Medical Affairs, MedRhythms, Inc., Portland, ME, USA staylor@medrhythms.com Louis N. Awad, PT, DPT, PhD (ORCID: 0000-0002-0159-8011) Department of Physical Therapy, Boston University, Sargent College of Health and Rehabilitation Sciences, Boston, MA, USA louawad@bu.edu Cecilia A. Carlowicz, MPH Department of Clinical Trials and Medical Affairs, MedRhythms, Inc., Portland, ME, USA ccarlowicz@gmail.com Yuri A. Maricich, MD, MBA (ORCID: 0000-0001-8667-5233) Independent physician-scientist, Cambridge, MA, USA yuri@maricich.org Seth P. Finklestein, MD (ORCID: 0000-0002-6682-3259) Stroke Service, Department of Neurology, Massachusetts General Hospital, Boston, MA, USA, sfinklestein@mgh.harvard.edu Erika H. Riley, MD, MPH Department of Medicine, Massachusetts General Hospital, Boston, MA, USA ehriley@mgh.harvard.edu Brian A. Harris (ORCID: 0009-0005-2803-8902) Department of Clinical Trials and Medical Affairs, MedRhythms, Inc., Portland, ME, USA bharris@medrhythms.com Ryan T. Pohlig, PhD (0000-0002-8385-8218) Biostatistics Core Facility, University of Delaware, Newark, DE, USA rpohlig@udel.edu Francois A. Bethoux, MD (ORCID: 0000-0003-4784-9418) Department of Physical Medicine and Rehabilitation, Cleveland Clinic Neurological Institute, Cleveland, OH, USA, bethouf@ccf.org *Corresponding author
Name and contact information for the trial sponsor {5b}	MedRhythms, Inc. 183 Middle Street, Suite 300, Portland, ME 04101 Contact: Sabrina Taylor, staylor@medrhythms.com
Role of sponsor {5c}	MedRhythms, Inc. is responsible for trial design, oversight, data interpretation, manuscript preparation, and funding, and provided the investigational device (MR‑001). MedRhythms delegated authority to a contract research organization to perform operational trial functions, including study coordination, monitoring, and data management.

## Introduction

### Background and rationale {6a}

Stroke is a leading cause of long-term disability worldwide [1], affecting over 7 million individuals in the USA alone [2]. Gait impairment is among the most prevalent and persistent consequences, impacting an estimated 54–80% of stroke survivors [3–5]. Such impairments compromise safety, limit independence, and reduce quality of life, particularly among older adults recovering from stroke [6–8]. While advances in acute stroke care have improved survival, they have also contributed to a higher long-term burden of disability. More individuals are now living with chronic post-stroke impairments. Direct medical costs are projected to exceed $50 billion annually and may more than double by 2035 [9, 10]. Walking difficulty is a primary concern for many survivors and their families [11]. These gait problems often co-occur with cognitive and emotional challenges that further restrict participation [10]. Scalable interventions that improve mobility and reduce healthcare utilization could help offset these rising costs while delivering value to patients, providers, and payers.

Access to high-quality gait rehabilitation remains limited, particularly for individuals in the chronic phase of recovery. Most interventions are resource-intensive, clinic-based, and poorly suited for long-term home use [12, 13]. As a result, many stroke survivors are left with few options for continued progress. Clinical guidelines increasingly call for scalable, personalized strategies to support mobility and reduce disability [14, 15]. Rhythmic auditory stimulation (RAS) is a well-validated intervention technique that engages auditory-motor entrainment (AME), a neurophysiological process in which the brain synchronizes movement to rhythmic auditory stimuli, such as in music [16, 17]. This synchronization can enhance gait neuromotor control by activating intact motor networks and potentially bypassing impaired pathways [18, 19].

RAS has been shown to improve walking speed, cadence, symmetry, and stride length post-stroke [20–23]. Walking and music-based interventions may also yield cognitive and emotional benefits, such as improvements in mood, memory, executive function, and quality of life [24–34]. However, most RAS studies have focused on short-term, supervised interventions [20]. Technology-enabled systems delivering RAS without the need for direct clinical supervision may offer a more scalable and sustainable model of care.

To address this gap, MedRhythms developed MR-001, an autonomous neurorehabilitation system that delivers individualized RAS in the home. A pilot study with an early prototype of MR-001 demonstrated that a fully automated, music-based RAS session reduced walking energy cost by ~9% and improved gait asymmetries by over 20% in individuals with chronic hemiparesis [35]. In a recent randomized controlled trial (RCT) [36] of 87 individuals with chronic post-stroke gait impairment (average >8 years post-stroke), participants who used MR-001 three times per week for 5 weeks improved walking speed by 22%, more than double the gain seen in controls (*p* = 0.013). Notably, 40% of MR-001 users exceeded the minimal clinically important difference (MCID) of 0.16 m/s [37]. They were also 3.7 times more likely to achieve both the MCID threshold and the community ambulation threshold of ≥0.8 m/s (*p* = 0.011). These findings demonstrate that stroke survivors many years after their initial event are still capable of achieving clinically meaningful gains with MR-001. Moreover, improvements continued to accrue over the 5-week intervention without evidence of plateau, supporting the potential for continued benefit with ongoing use.

The current longitudinal study builds on these previous findings, integrating both pragmatic and traditional controlled trial elements to evaluate the MR-001 intervention in a broader eligible population, over an extended intervention duration, and including follow-up and re-treatment phases in a real-world setting to assess the durability and replicability of benefit. Critically, it prioritizes engagement—measured as the proportion of participants achieving at least moderate engagement over the 12-week intervention period—as the primary endpoint, recognizing that consistent use is essential for durable, long-term impact. The study, therefore, aims to characterize real-world adherence and usage patterns of home-based RAS in daily life. In parallel, the study includes exploratory analyses to assess the potential economic impact of MR-001 by linking clinical outcomes with healthcare claims data. Indeed, stroke-related disability is a major cost driver, often leading to falls, hospitalizations, and long-term care. A prior budget impact model projected substantial cost savings with MR-001 [38], but empirical data are lacking. This trial will explore whether mobility improvements with MR-001 might correspond to reduced healthcare utilization over 12 months, offering early insights to support cost-effectiveness evaluations and the role of digital neurorehabilitation in value-based stroke care.

### Objectives {7}

This study aims to evaluate user patterns of engagement, clinical effectiveness, durability of response, and health economic impact on participants post-stroke who complete the MR-001 intervention in the home setting. The primary objective is to assess engagement with the MR-001 neurorehabilitation system, measured by the proportion of participants achieving at least moderate engagement over the 12-week intervention period. The secondary objectives are to evaluate: (1) the effects of MR-001 on walking endurance, as measured by the 6-Minute Walk Test (6MWT); (2) the durability of this effect following 12 weeks of intervention; and (3) the effects of MR-001 on health-related quality of life, activities of daily living, social isolation, and cognition and executive function. Exploratory objectives include assessing: (1) changes in general mobility, as measured by the Timed Up and Go Test (TUG); (2) the effects of a second period of treatment on walking endurance and general mobility following a 3-month no-intervention period; (3) the differential intervention effects across subgroups (e.g., engagement and baseline gait impairment subgroups); (4) the proportion and predictors of responders; and (5) healthcare resource utilization, assessed through claims data analysis.

### Trial design {8}

This longitudinal, pragmatic, decentralized hybrid trial integrates home-based implementation of the MR-001 therapy with selected controlled trial elements to maximize external validity while maintaining scientific rigor. In this study, “home-based” refers to any environment outside of a professional healthcare facility consistent with the FDA definition [39]. A two-step, withdrawal with randomized re-treatment (delayed randomization) design allows assessment of treatment engagement, clinical outcomes, durability of benefit, and the effects of re-treatment in the home setting. This hybrid design allows for evaluation of both immediate and sustained effects of the intervention, as well as the potential benefit of reintroduction following initial use. Spanning ~52 weeks, the study follows a decentralized design without traditional clinical trial sites. In-person assessments are conducted at more than 30 designated physical assessment center locations across the USA, chosen to ensure broad geographic representation of the population.

Step 1: All participants receive 12 weeks of MR-001 intervention (30 min per session, three times per week), followed by a 12-week washout. The 12-week intervention period was selected based on (1) extending the established evidence that users do not plateau after 5 weeks of MR-001 intervention [36] and (2) stroke rehabilitation evidence supporting this duration as effective for achieving clinically meaningful gains in walking capacity [40]. This timeframe also aligns with guidelines recommending sustained, task-specific therapy to support neuroplasticity and motor learning [14], and reflects typical US outpatient rehabilitation reimbursement models, which frequently authorizes approximately 36 therapy sessions over 12 weeks [41]. Participants self-administer MR-001 in their home environment, with remote support provided by the study team throughout the 12-week intervention period.

Step 2: At the end of the washout, participants are randomized 1:1 to either receive the MR-001 intervention for another 12 weeks followed by 12 weeks of washout, or continue with an additional 24-week washout. Participants and investigators are unblinded to the Step 2 allocation, but in-person assessors remain blinded.

**Fig. 1:**
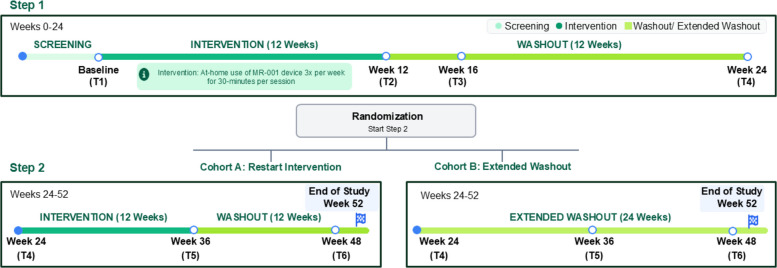
Study design

Assessments occur at baseline and weeks 12, 16, 24, 36, and 48, with continuous falls/adverse event tracking and healthcare resource utilization (HRU) data collection over ~52 weeks. Collected outcomes include gait performance (6MWT), patient-reported outcomes (Barthel Index [BI; Step 1 only], PHQ-8, PROMIS Social Isolation), and cognitive tests (Trail Making Test A and B [TMT A and B; baseline and week 12 only]). Figure 1 illustrates the study design.

## Methods: participants, interventions, and outcomes

### Study setting {9}

**Fig. 2:**
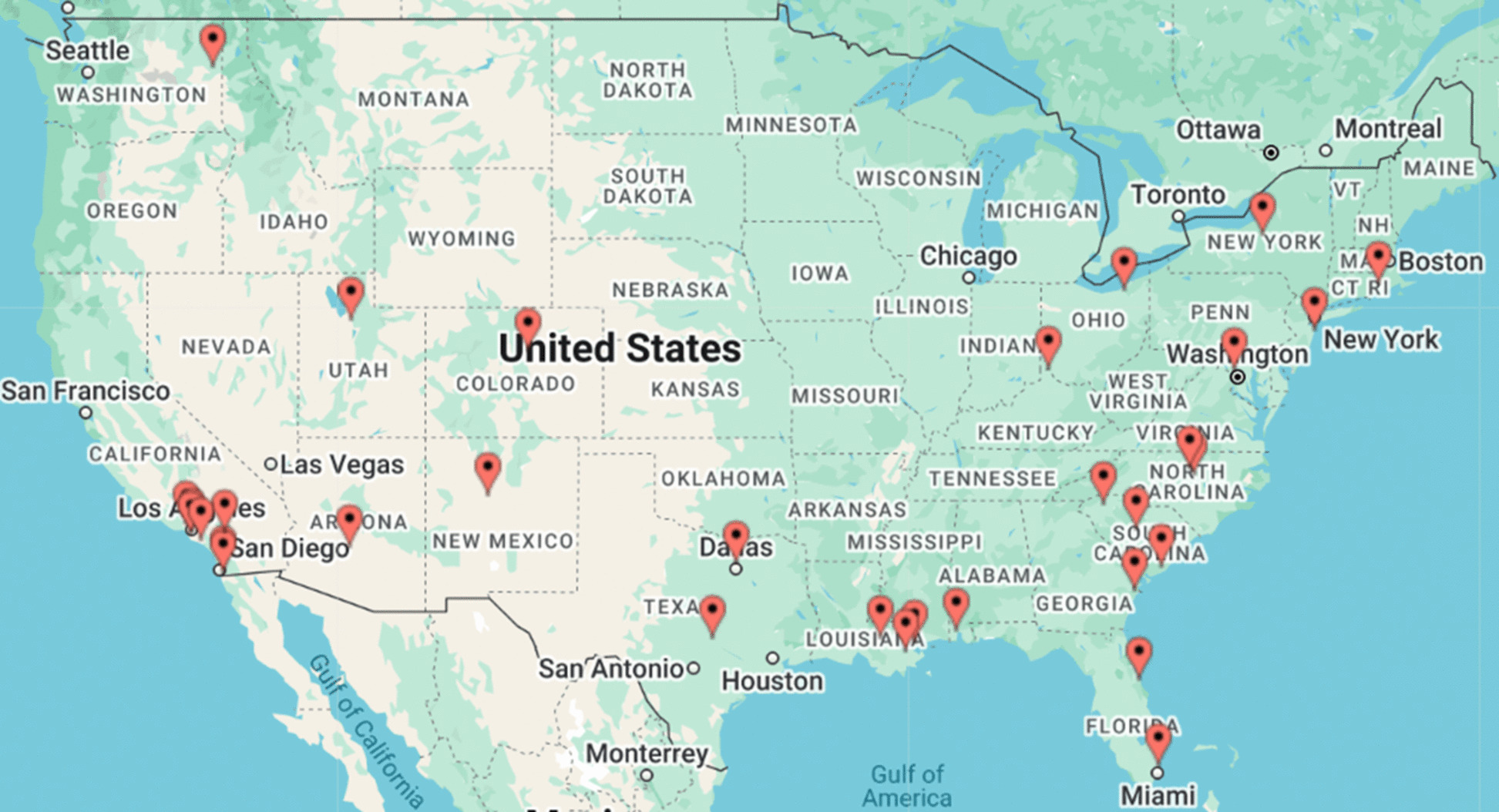
Assessment center locations across the USA. Image generated using batchgeo.com

This decentralized, US-based clinical trial uses designated physical assessment centers—primarily through Velocity Clinical Research and Advanced Clinical Institute—for in-person gait performance assessments and clinical evaluations. Consistent with FDA guidance on decentralized trials [42], these locations serve exclusively as assessment venues and do not operate as investigational sites of record; personnel at these centers are not considered study staff, and they have no principal investigator, on-site study team, or regulatory responsibilities. At study initiation in October 2023, 30 such assessment centers were trained and launched. Assessment centers were strategically selected across US regions to ensure broad geographic representation. Assessment center selection began with 57 potential locations. Each location was evaluated using demographic and epidemiologic criteria, including population characteristics (city, state, county), age distribution, stroke prevalence and incidence (CDC data), covered lives by state (Flexpa database, including Medicare), and population diversity (race/ethnicity, languages spoken). Assessment centers with overlapping geographies were excluded. Figure 2 illustrates the locations of the selected assessment centers.

Intervention sessions are self-administered in the home setting, supported remotely by the Contract Research Organization (CRO), Curavit Clinical Research, via secure digital platforms and teleconsultations.

### Eligibility criteria {10}

#### Inclusion criteria (all must be met)

- Equal to or greater than 6 months post-stroke with gait impairment
- Age 18–85 years inclusive
- Understand and speak English
- Able to ambulate without assistance from another person (assistive devices allowed and must be used consistently)
- Willing to travel to a clinical testing location to complete in-person assessments
- Able to walk at a speed greater than or equal to 0.4 m/s* on 6-Minute Walk Test
  *Walking speed criterion selected to ensure adequate performance of MR-001

- Must have claims data available and consent to sharing

#### Exclusion criteria (any of the following)

- Hearing impairment such that the participant cannot hear the rhythmic stimulation of the music
- Pain that impairs walking ability
- Unable to safely participate in walking sessions as determined by the investigator
- Requires more than one rest during the 6-Minute Walk Test
- Significant comorbid medical or neurological conditions that could impact gait or prevent safe participation (e.g., Parkinson’s disease, cerebral palsy, multiple sclerosis, myasthenia gravis, muscular dystrophy, spinal cord injury, recent major surgery within 3 months)
- Pregnant or become pregnant during the study
- Lower limb prosthetic
- More than 2 falls in the previous month
- Non-reciprocal gait pattern (must have a 2-point step pattern)
- Treatment with a gait-based investigational intervention within the last 3 months
- Unable or unwilling to provide informed consent

### Who will take informed consent? {26a}

Informed consent is obtained remotely, and electronically, by qualified study personnel who have received appropriate training in Good Clinical Practice (GCP) and the specific requirements of this protocol. All individuals obtaining consent must be listed on the delegation log and have documented training in the informed consent process.

### Additional consent provisions for collection and use of participant data and biological specimens {26b}

No biological specimens are collected in this study. Participants provide specific consent for the collection and use of healthcare resource utilization data from insurance claims databases. Participants also consent to the use of de-identified data for future research purposes and potential sharing with regulatory authorities and scientific community through publications and presentations.

## Interventions

### Explanation for the choice of comparators {6b}

This study uses a withdrawal with randomized re-treatment design. The rationale for this design includes:

1. Established safety and efficacy: A prior RCT demonstrated that MR-001 leads to superior improvements in walking outcomes compared to unassisted walking without increasing adverse events [36]. These results provide strong evidence of efficacy and safety, making a full RCT unnecessary for Step 1 of this trial. Instead, all participants receive the intervention to establish patterns of real-world user engagement and adherence, which is the primary endpoint of interest in this pragmatic study.
2. Ethical considerations: Given the potential benefits of RAS therapy and the limited access to such interventions for chronic stroke patients, it was deemed more ethical to provide all participants the opportunity to receive the intervention.
3. Implementation evidence: The design allows for assessment of intervention durability and the impact of treatment interruption in an approximation of a real-world setting, providing valuable insights for implementation.

### Intervention description {11a}

MR-001 (MedRhythms, Inc., Portland, ME, USA) is an autonomous neurorehabilitation device that administers RAS. MR-001 is commercially available in the USA as a prescription medical device under the brand name, InTandem®, for physical rehabilitation of ambulatory adults with chronic stroke walking impairments. For a detailed overview of the MR-001/InTandem neurorehabilitation system, see Awad et al. [36]. In brief, the system continuously assesses the user’s entrainment to the target tempo and evaluates gait symmetry and variability, while safely and autonomously adjusting the target walking speed without direct clinician input. The system consists of:

-
  - Shoe-worn inertial sensors: Two shoe-worn wearable inertial sensors that measure walking patterns and gait parameters in real time
  - Touchscreen control unit: A kiosked (locked) touchscreen control unit preloaded with autonomous intervention software that processes gait data and delivers personalized therapy
  - Headset: Audio delivery system
  - Charging equipment: For maintaining device functionality
  - Instruction for Use (IFU) manual: Physical booklet containing detailed guidance on the proper operation, safety warnings and precautions, and clinical application of MR-001 to ensure safe and effective use

Participants use MR-001 for 30 min per session, three times per week, for a total of 12 weeks (approximately 36 sessions). The walking program is conducted independently in the home environment. The system provides a personalized intervention, with proprietary, adaptive algorithms that progress the therapy based on participant gait patterns and biomechanics.

While the MR-001 device is commercially available in the USA, it is distributed for investigational use in this study to evaluate additional clinical outcomes distinct from those in the commercial device’s current labeling. The device has been determined to be non-significant risk according to FDA criteria (21 CFR 812.3(m)), as it is not an implant, does not support or sustain life, and does not represent substantial risk to participant health or safety. The study, therefore, met abbreviated requirements for investigational device exemption (21 CFR 812.2(b)(1)).

### Criteria for discontinuing or modifying allocated interventions {11b}

Modification to the duration and/or frequency of the MR-001 intervention is not pre-specified in the protocol. Participants may discontinue the intervention based on the following criteria:

- Participant request to withdraw from the intervention
- Adverse event that poses safety risks or significantly impacts participant well-being
- Investigator determination that continued participation poses safety risks
- A change in participant medical status that results in the participant meeting an exclusion criterion that contraindicates safe participation

Each walking session with MR-001 should be completed within the required 30-min timespan and should not be split throughout the day. Participants may elect to complete more than three sessions per week during the intervention phase, if discussed with study investigators and no safety concerns are raised.

### Strategies to improve adherence to interventions {11c}

Several strategies are employed to optimize participant adherence:

- Onboarding support: Participants receive training on device use and study expectations via a guided setup call upon receipt of the device in their home. This simulates the education a patient might receive from a provider when obtaining a prescription.
- Flexible scheduling: Participants can choose their preferred times and locations for walking sessions within safety guidelines (e.g., not on a treadmill or busy streets) as noted in the protocol and described in the participant-facing Instruction for Use manual that is provided to all participants with the MR-001 system.
- Technical support: Access to a dedicated technical support hotline for device-related issues, which mirrors the optional support available from the manufacturer (MedRhythms, Inc.) for commercialized devices.
- Regular contact and reminder systems: Scheduled check-ins with study staff to address questions and provide support. Weekly engagement emails sent during the walking phase. Optional text/email reminders for appointments. Check-in calls when a participant has not walked for at least 7 days.

### Relevant concomitant care permitted or prohibited during the trial {11d}

#### Permitted concomitant care

- Standard medical care and medications as prescribed by treating physicians
- Physical therapy and occupational therapy (must be documented)
- Use of assistive devices for mobility (must remain consistent throughout the study)
- Participation in general exercise programs not specifically targeting gait

#### Prohibited concomitant care

- Participation in other investigational interventions targeting gait or mobility
- Initiation of new intensive gait-specific rehabilitation programs during the study period
- Use of other gait-based treatments or similar interventions

### Provisions for post-trial care {30}

No specific post-trial care is provided by the sponsor, but participants are encouraged to discuss ongoing rehabilitation needs with their healthcare providers. If participants request information about the commercial availability of MR-001, they are sent an email with an attached InTandem Information Kit and links to the InTandem website [43] and an informational video.

### Outcomes {12}

The primary endpoint is participant engagement with the MR-001 neurorehabilitation system, operationalized as the proportion of participants achieving at least moderate engagement over the 12-week treatment phase. Engagement will be categorized as low, moderate, or high based on predefined thresholds of weeks, sessions, and minutes of use, with the proportion of participants achieving at least moderate engagement defined by exceeding a benchmark of 60%. Session completion will be recorded automatically by the device’s usage logs, with higher counts indicating greater adherence to the prescribed rehabilitation regimen.

Secondary endpoints include:

- Walking endurance: Change in distance walked on the 6MWT [44] from baseline to the end of the 12-week intervention. The 6MWT measures the maximum distance an individual can walk on a flat surface in 6 min, with greater distances reflecting improved endurance and mobility.
- Durability of response: Change in 6MWT distance from the end of the intervention to the end of the subsequent washout period, to assess whether mobility gains are maintained without ongoing treatment.
- Depressive symptoms: Change in scores on the Patient Health Questionnaire-8 (PHQ-8) [45], an 8-item self-report measure of depressive symptom severity (score range: 0–24; higher scores indicate more severe symptoms).
- Activities of daily living: Change in the BI [46] score, which rates independence in basic daily activities such as feeding, bathing, dressing, and mobility (score range: 0–100; higher scores indicate greater independence).
- Social isolation: Change in scores on the PROMIS Social Isolation Scale [47, 48], which assesses perceived isolation from others, with higher T-scores indicating greater isolation.
- Cognitive function: Change in completion time for TMT Parts A and B [49, 50], neuropsychological measures of processing speed (Part A) and executive function/set-shifting (Part B), where shorter completion times indicate better performance.

Exploratory endpoints include:

- 6MWT results collected during the re-treatment phase (Step 2 of the study).
- TUG [51] test results in both Step 1 and Step 2, assessing functional mobility by timing the participant rising from a chair, walking 3 meters, turning, returning, and sitting (shorter times indicate better mobility).
- Subgroup analyses: Assessing differential intervention effects on primary, secondary, and exploratory outcomes across engagement levels and baseline gait impairment.
- Clinical responsiveness: Assessing the proportion of responders (i.e., participants who achieve at least the minimal clinically important difference (MCID) on the 6MWT) to help quantify the proportion of patients experiencing a clinically meaningful benefit.
- Predictors of response: To assess which subgroups may benefit most from the intervention, baseline demographic and clinical characteristics, as well as engagement levels with the device, will be examined as potential predictors of responder versus non-responder status.
- Healthcare resource utilization assessed through claims data analysis, including total all-cause healthcare encounters (outpatient visits, emergency department visits, and hospitalizations) and the proportion of participants with all-cause emergency department visits and hospitalizations from baseline through ~52 weeks.

### Participant timeline {13}

After providing informed consent, participants complete screening assessments within 28 days prior to baseline, including demographic and medical history (with past falls), eligibility confirmation, and in-person testing with the 6MWT, TUG, and TMT A and B.

Baseline assessments are conducted within 14 days before starting the intervention and include the PHQ-8, PROMIS Social Isolation Scale, BI, and falls/adverse event documentation. Participants then complete a 12-week intervention phase with three MR-001 walking sessions per week, supported by engagement calls. Primary and secondary outcomes are reassessed at the end of week 12. Follow-up visits at weeks 16 and 24 evaluate maintenance of effects during the washout period.

Randomization occurs at the end of week 24, assigning participants to either cohort A, which receives the MR-001 intervention for another 12 weeks (weeks 25–36), or cohort B, which continues washout. Outcome assessments are repeated at weeks 36 and 48. Claims re-authorization is performed, as needed, and healthcare utilization data are collected throughout the study with a final follow-up call conducted at approximately week 60 to ensure collection of 52 weeks of HCRU data. The participant timeline detailing the schedule of enrollment, interventions, and assessments is illustrated in Table 1.

**Table 1:** Participant timeline

	Step 1						Step 2			
	Screening	Baseline (T1)	Intervention Step 1	Post- Intervention (T2)	Follow Up 1 (T3)	Follow-Up 2 (T4)	Intervention Step 2/Cont. Washout	Follow-Up 3 (T5)	Follow-Up 4 (T6)	Follow-Up call (as needed)
**Timepoint**	Day −28 to day 0	Day −14 to Day 0	Weeks 1–12	End of week 12 (+/- 7 days)	End of week 16 (+/- 7 days)	End of week 24 (+/- 7 days)	Weeks 25 - 36	End of week 36 (+ 14 days)	End of week 48 (+ 28 days)	Through Week 60
**ENROLLMENT**										
Informed consent	X									
Demographics & Med hx	X									
Eligibility screen	X									
Claims data consent	X									X
Randomisation						X				
**INTERVENTIONS**										
MR-001 (3x/week)			X				X (Cohort A only)			
**ASSESSMENTS**										
6-Minute Walk Test	X			X	X	X		X	X	
Timed Up and Go	X			X	X	X		X	X	
Trail Making Test A & B	X			X						
Patient Health Questionnaire-8		X		X	X	X			X	
PROMIS Social Isolation		X		X	X	X			X	
Barthel Index		X		X	X	X				
Falls/AE collection		X	X	X	X	X	X	X	X	X

### Sample size {14}

The target enrollment is 225 participants, allowing for an anticipated 20% attrition to yield approximately 180 participants completing the study. The calculation is based on the primary endpoint of device engagement, tested using a one-sided binomial proportion test with alpha = 0.05 against a null hypothesis of 60% engagement. Assuming a true engagement rate of 72%, this sample size provides 98% power to reject the null. Exploratory analyses will also assess engagement thresholds of 70% and 80%.

### Recruitment {15}

Participants are identified through a multi-step, IRB-approved strategy combining digital outreach, clinical referrals, and community engagement. The primary method of recruitment is via targeted digital advertising within ~50 miles of assessment center, distributed through multiple recruitment vendors, including 1nHealth, Goodlab, 1Digital, and Clinical Connection. Referrals also come from stroke specialists, rehabilitation providers, patient advocacy groups, support networks, and, where permitted, healthcare system databases. While recruitment methods are focused on a 50-mile radius from a physical assessment center, participants are not excluded from the study in the pre-screener if they live at a greater distance as described further below.

All referred individuals are directed to a centralized study landing page that contains general study information, frequently asked questions (FAQs), a pre-screener link, and gait demonstration videos. Interested individuals complete an online pre-screener, allowing them to self-attest to key inclusion and exclusion (I/E) criteria. Individuals who screen out due to being less than 6 months post-stroke are flagged for re-contact at a later date. During the pre-screener, participants are provided with a list of all physical assessment locations and asked if they are willing to travel to one. If they decline, they are disqualified; if they agree, regardless of proximity, they may proceed to the eligibility call.

Participants meeting pre-screener criteria are invited to an eligibility call with the study team. Real-time call initiation is supported, along with the ability to schedule calls with study staff within the following few weeks. Participants receive automated reminders before scheduled calls, and coordinators proactively reach out to candidates to support scheduling. During the eligibility call, trained team members confirm I/E criteria and initiate the informed consent process.

Eligible participants then attend an in-person screening visit at a physical assessment location. Data and source documentation collected during in-person screening are uploaded into CRIO, including walking videos to determine walking safety. Study investigators review all submitted materials to make the final eligibility determination. Those deemed eligible move forward to baseline visit planning, which includes scheduling of the baseline assessment and a guided setup call. The study kit is shipped from the distribution center (C3i Solutions, Pittston, PA) to arrive ~3–5 days before the guided setup call.

This stepwise, digitally coordinated recruitment strategy, with evolving process improvements and human support, is designed to minimize friction, support participant comfort, and promote successful enrollment in a decentralized study design.

#### Mid-study adaptations

After study initiation, trial monitoring revealed an opportunity for several operational refinements to ensure successful completion of the trial objectives. These included assigning a dedicated participant coordinator to reduce missed eligibility calls, allowing informed consent and claims authorization to be completed in separate calls based on participant preference, expanding the number of assessment centers from 30 to 33 to increase geographic reach and increase recruitment capacity, and refining outreach workflows to enhance scheduling efficiency. All adaptations were made in response to observed challenges with trial operation and based on participant feedback. These changes did not alter the study objectives, endpoints, or analytic plan.

## Assignment of interventions: allocation

### Sequence generation {16a}

Randomization for Step 2 is performed using Python’s built-in random shuffle function. Participants are randomized in a 1:1 ratio to either receive another 12 weeks of MR-001 intervention (cohort A) or continue washout for an additional 24 weeks (cohort B).

### Concealment mechanism {16b}

The randomization sequence is generated by the CRO and implemented through Ripple Science (Ann Arbor, MI), the Clinical Trial Management System (CTMS) for this trial. Site personnel do not have access to the randomization sequence prior to participant allocation. Randomization assignments may be revealed only after all Step 1 assessments are completed and data are entered into the system.

### Implementation {16c}

Eligible participants who complete Step 1 are randomized at the week 24 visit. The CTMS automatically assigns participants to their allocated cohort after completion of all required week 24 assessments. Site personnel communicate the assignment to participants and initiate the appropriate Step 2 procedures.

## Assignment of interventions: blinding

### Who will be blinded {17a}

This is an open-label study during Step 2. Participants and investigators are unblinded to cohort assignment following randomization. However, outcome assessors conducting in-person gait assessments are blinded to cohort assignment. Data analysts remain blinded to treatment assignment until database lock.

### Procedure for unblinding if needed {17b}

As this is an open-label study during the randomized phase, formal unblinding procedures are not applicable. However, in cases where knowledge of previous intervention exposure is critical for safety decisions, complete treatment history is available to investigators for review.

## Data collection and management

### Plans for assessment and collection of outcomes {18a}

Data collection occurs through multiple mechanisms:

- In-person assessments: Conducted at clinical research assessment centers for gait performance assessments (6MWT, TUG) and cognitive assessments (TMT A and B). Trained personnel conduct assessments according to standardized protocols.
- Remote assessments: Patient-reported outcomes (PHQ-8, BI, PROMIS Social Isolation Scale) are collected electronically through secure platforms.
- Device data: The MR-001 system automatically captures session data, adherence metrics, and technical performance indicators.
- Healthcare utilization data: Claims data is obtained from insurance providers and healthcare systems with appropriate participant authorization and data use agreements through Flexpa.

### Plans to promote participant retention and complete follow-up {18b}

To promote participant retention and ensure complete follow-up, the study implements several engagement strategies. These include regular communication and personalized support from study staff, as well as flexible scheduling options to accommodate participants’ availability. In addition, weekly emails are sent while a participant has MR-001 to increase engagement touchpoints. Participants receive compensation for their time and travel expenses. Additionally, travel support to all in-person visits via study staff-scheduled Lyft or Uberhealth rides is provided for those who request the additional support.

### Data management {19}

Data is collected and managed using Clinical Research IO (CRIO), a 21 CFR Part 11-compliant electronic source and electronic data capture (eSource/EDC) system. During in-person assessments, staff performing the physical assessments capture the data using paper source, which is then entered into CRIO. The paper source documentation is also uploaded into CRIO for source data verification (SDV) to ensure the data entered matches the source documentation. Any discrepancies are queried with the site. Data collected during remote assessments is entered directly into CRIO without paper source. Built-in edit checks and role-based access controls ensure data accuracy, integrity, and security. All data entries are time-stamped and audit-trailed to facilitate regulatory compliance. Source document verification is performed according to the monitoring plan.

Data quality is ensured through:

- Real-time data validation and query generation
- Regular data review meetings
- Source data verification
- Double data entry for critical variables
- Automated range and consistency checks

The study design includes a phased database lock strategy, with multiple database locks planned following completion of key study timepoints. Specifically, database locks will occur after all participants have completed their 3-month, 6-month, and final study visits. This approach supports timely data dissemination while preserving data integrity.

Each database lock will occur only after all data queries have been resolved and all monitoring activities completed for the relevant study visit. As all data required for each pre-specified endpoint will have been collected prior to lock, no adjustments to the planned alpha level will be necessary for the corresponding analyses. Changes to the database after lock will not be permitted unless predefined unlock criteria are met and appropriately documented.

### Confidentiality {27}

All study data is handled in accordance with applicable privacy regulations, including HIPAA. Participants are assigned unique study identification numbers, and all data is de-identified for analysis purposes. Access to identifiable information is limited to authorized study personnel with documented training in data privacy and confidentiality.

Data sharing agreements are established with all parties handling participant data, including technology vendors and data analysis contractors. All electronic data will be encrypted and stored on secure servers with appropriate backup and disaster recovery procedures.

### Plans for collection, laboratory evaluation, and storage of biological specimens for genetic or molecular analysis in this trial/future use {33}

n/a—No biological specimens are collected in this study.

## Statistical methods

### Statistical methods for primary and secondary outcomes {20a}

Primary analysis: The primary endpoint is engagement with MR-001, defined as the proportion of participants achieving at least moderate engagement. Engagement will be categorized as low, moderate, or high based on predefined thresholds of weeks, sessions, and minutes of use. The primary hypothesis is that the proportion of participants achieving at least moderate engagement exceeds a benchmark of 60%. Sensitivity analyses will be conducted at thresholds of 70% and 80%. A one-sided binomial proportion test will be used at significance level *α* = 0.05. Participants who initiate the intervention but withdraw from the study prior to completion of the planned intervention will be classified as non-engaged in the primary analysis.

Secondary analyses: Secondary endpoint analyses will evaluate mean changes from baseline to post-intervention for walking endurance (6MWT), depressive symptoms (PHQ-8), independence in activities of daily living (BI), social isolation (PROMIS Social Isolation Scale), and cognitive and executive function (TMT A and B).

Durability of walking endurance will be assessed at 16-week and 24-week follow-up visits after discontinuation of the intervention at 12 weeks. Results at these follow-up visits will be compared with the post-intervention assessment to determine whether the gains achieved during treatment are sustained. The analysis will be conducted using a non-inferiority framework, with a pre-specified margin of one-half of the minimal clinically important difference (17.2 m) [52]. This approach is designed to test whether the benefits observed during the intervention period are retained after device use is discontinued. Linear mixed modeling (LMM) will be used to estimate the changes over time. Random intercepts and/or modeling the covariance matrix of the error terms will be used to account for repeated measures. Model choices will be made based on minimizing Akaike Information Criteria (AIC) and Bayesian Information Criteria (BIC). Age, sex, baseline speed, and time since stroke will be evaluated and included as covariates if they are predictive of the change scores.

### Interim analyses {21b}

A single interim analysis for futility will be conducted when approximately 50% of participants have completed Step 1. The analysis will focus on the primary engagement endpoint and safety data. The study may be terminated early if engagement rates are substantially lower than expected or if safety concerns arise. No formal stopping rules for efficacy are planned, as this would require adjustment of the overall alpha level.

### Methods for additional analyses (e.g., subgroup analyses) {20b}

#### Exploratory analyses

Exploratory analyses are planned to provide additional insight into the effects of MR-001 and to identify factors that may influence treatment response. These will include:

- Change in general mobility (TUG): Mobility will be assessed at multiple study visits across the intervention, follow-up, and re-treatment phases. Analyses will explore whether the MR-001 intervention is associated with sustained or progressive improvements in functional mobility, as well as whether gains are maintained after withdrawal or re-established with re-treatment.
- Clinical responsiveness: Participants who achieve at least the minimal clinically important difference (MCID) on the 6MWT will be classified as responders. This analysis will help quantify the proportion of patients experiencing a clinically meaningful benefit, complementing the mean change analysis.
- Predictors of response: Baseline demographic and clinical characteristics, as well as engagement levels with the device, will be examined as potential predictors of responder versus non-responder status. This analysis is intended to inform which subgroups may benefit most from the intervention.
- Durability of re-treatment: Among participants who undergo a second round of intervention following the withdrawal phase, changes in walking mobility and endurance will be compared before and after re-treatment to assess whether the intervention effect can be re-established.

#### Health economics analyses

In addition, the study will explore the impact of MR-001 on healthcare resource utilization (HRU) among participants. The feasibility of this analysis depends on (1) adequate long-term retention of participants, (2) successful linkage of participants to claims data, and (3) availability of funding. If all conditions are met, analyses will proceed as described below; otherwise, the HRU analysis will not be conducted (go/no-go criterion). Depending on data availability and funding resources, analyses could include an external control arm (ECA), matched to trial participants on key baseline characteristics, developed from electronic health records or claims databases, or deploy a within-subject design where HRU will be compared before and after MR-001 enrollment within participants, using appropriate longitudinal methods.

The planned economic analyses are subject to refinement to ensure alignment with available data and evolving methodological standards. A detailed Health Economics Analysis Plan (HEAP) will be finalized prior to analysis.

### Methods in analysis to handle protocol non-adherence and any statistical methods to handle missing data {20c}

Primary analysis will follow an intention-to-treat (ITT) approach, including all randomized participants in their assigned groups (for Step 2) regardless of adherence or protocol deviations. A per-protocol (PP) analysis will also be performed, excluding participants with major protocol violations.

Missing data will be handled under the missing at random (MAR) assumption. For the primary endpoint, participants who initiate the intervention but discontinue before completion will be classified as non-engaged. For secondary and exploratory outcomes, longitudinal analyses will use linear mixed models (LMM), which inherently accommodate incomplete follow-up data without the need for additional imputation by maximum likelihood estimation. This approach includes all available observations while appropriately addressing missingness conditional on observed data.

### Plans to give access to the full protocol, participant-level data, and statistical code {31c}

The full study protocol will be made publicly available through trial registration platforms and journal publication. De-identified participant-level data and statistical analysis code will be made available to qualified researchers upon reasonable request and approval by the study sponsor, following completion of the primary analyses and publication of main results. Data sharing will comply with applicable privacy regulations and institutional policies. Requests for data access should be directed to the corresponding author and will be evaluated by a data access committee including the principal investigator and sponsor representatives.

## Oversight and monitoring

### Composition of the coordinating center and trial steering committee {5d}

#### Coordinating center

Curavit Clinical Research serves as the coordinating center for this trial, providing comprehensive operational and technical support. As the Contract Research Organization (CRO), Curavit is responsible for the design and maintenance of Ripple Science as the overarching CTMS, which includes the implementation of randomization in Step 2, and CRIO, the eSource/EDC system deployed for this trial. They oversee data quality and integrity through centralized monitoring and support real-time data review and query resolution. Curavit also coordinates the distribution of the MR-001 device to participants via their distribution partner, C3i. In addition, Curavit manages study logistics, facilitates communication among study stakeholders, supports remote and site-based assessments, and ensures adherence to regulatory requirements and GCP. Through these efforts, Curavit plays a central role in maintaining trial compliance, participant safety, and operational efficiency. Curavit holds weekly operational meetings with the sponsor to review enrollment, logistics, safety data, and protocol adherence.

#### Trial steering committee

The Scientific Steering Committee is comprised subject-matter experts appointed based on their qualifications, experience, and relevance to the scientific and clinical domains addressed by the study. The Scientific Steering Committee is responsible for oversight of the trial’s scientific integrity and operational execution. Its duties include providing strategic input into the trial design, reviewing and approving the study protocol and related documents, and supervising overall trial conduct. The committee convenes on an as-needed basis to evaluate study progress, monitor safety data, and review any proposed protocol amendments.

#### Principal and co-investigators

The principal investigator (PI) is a member of the Scientific Steering Committee and is responsible for the overall conduct of the trial, including ensuring compliance with the protocol, GCP, and applicable regulatory requirements. Key responsibilities include overseeing participant safety and informed consent, study staff, data accuracy and source documentation, investigational device accountability, and timely reporting of adverse events and protocol deviations. The PI retains ultimate accountability for trial integrity, staff training, and regulatory correspondence throughout the study lifecycle. There are two sub-investigators (Sub-Is) on this trial supporting the PI. The Sub-Is are qualified by education, training, and experience. While the PI is ultimately responsible for all study conduct, the Sub-Is provide additional medical oversight and support the PI with completion of study activities that require higher levels of medical training. This includes review of medical history, concomitant medications, and study documentation to determine eligibility. They also are responsible for evaluating participant safety during the study including review of adverse events and ensuring proper documentation and reporting of AEs/SAEs.

### Composition of the data monitoring committee, its role and reporting structure {21a}

n/a—A data monitoring committee is not convened for this trial, as the study involves a minimal-risk intervention and does not include investigational products or procedures warranting external safety oversight. Ultimately, the PI provides primary safety oversight with additional oversight of participant safety through a formal Data and Safety Monitoring Plan (DSMP), which outlines predefined internal procedures for safety surveillance, adverse event review, and protocol compliance. These procedures are implemented by the study team and sponsor, with regular monitoring activities to ensure adherence to regulatory and ethical standards.

### Adverse event reporting and harms {22}

During every study visit, study staff ask participants if they have had any changes to their health, and document any adverse events (AEs) in CRIO. All AEs are recorded in the AE log where study staff can electronically request a co-investigator’s review. The co-investigator determines severity, seriousness, and relationship to the study intervention. Serious adverse events are reported to the sponsor and PI within 24 h of awareness.

Expected adverse events related to the intervention include:

- Fatigue
- Muscle or joint soreness
- Nausea
- Shortness of breath
- Minor muscle cramps
- Pain
- Dizziness
- Loss of balance or fall
- Skin irritation (from sensors/headset)
- Bruising and bleeding
- Muscle strain/tear
- Injury due to fall (ankle sprain, twisted knee, etc.)
- Abnormal physiological response to physical activity (e.g., irregular heart rate, fainting, etc.)

These events are anticipated based on prior clinical experience, the device’s mechanism of action, and the underlying chronic stroke condition being studied. They are not considered acceptable or unimportant, but rather recognized as foreseeable risks that will be closely monitored. In this study, falls are considered adverse events of special interest (AESIs) and are reported promptly to the sponsor and PI with detailed documentation.

All adverse events will be followed until resolution or stabilization. Annual safety reports will be prepared and submitted to regulatory authorities and ethics committees, as required.

### Frequency and plans for auditing trial conduct {23}

Trial conduct will be audited through virtual monitoring activities conducted by a designated study monitor. The study monitor will review the available study data at approximately four times throughout the course of the trial. An initial safety monitoring review will occur within approximately 30 days of the first participant receiving the study device. Subsequent audits will be conducted at key milestones: (1) when approximately 50% of participants are enrolled in Step 1, (2) after the first 10% of successful randomizations in Step 2, and (3) upon completion of Step 2. Additional reviews may be combined or adjusted based on the data collection timeline. The monitor will review participant safety, protocol compliance, data quality, and adherence to GCP. Key risk indicators (e.g., AE/SAE/AESI rates, withdrawals, protocol deviations, or compliance issues) may trigger more frequent reviews. Monitoring reports will be generated after each review, and any necessary corrective actions will be documented and reviewed by the PI and sponsor.

### Plans for communicating important protocol amendments to relevant parties (e.g., trial participants, ethical committees) {25}

All proposed protocol amendments are reviewed first by the sponsor and PI. Substantial amendments are submitted to the IRB and regulatory authorities for approval before implementation. After IRB approval, the CRO distributes the updated protocol and summary of changes to all investigators and assessment center personnel. Participants will be reconsented when changes impact participant rights or safety. ClinicalTrials.gov will be updated promptly following the implementation of any amendment affecting registered fields. Administrative amendments may be implemented immediately with retrospective notification.

### Dissemination plans {31a}

Study results will be disseminated through multiple channels, including publication in peer-reviewed journals, presentation at scientific conferences, and submission to relevant regulatory authorities. Findings will also be shared with patient advocacy organizations, posted as updates to clinical trial registries, and communicated through press releases and media outlets as appropriate. Authorship of resulting publications will be determined in accordance with the guidelines established by the International Committee of Medical Journal Editors (ICMJE).

## Discussion

Building on established evidence, this longitudinal, pragmatic, decentralized clinical trial evaluates MR-001, a technology-enabled, autonomous neurorehabilitation system that delivers individualized RAS in the home. By combining home-based intervention delivery, extended intervention and follow-up periods (Step 1), a randomized re-treatment phase (Step 2), and integration of both clinical and economic outcomes, this study aims to provide evidence that is directly relevant to patients, clinicians, payers, and health systems. While prior studies have shown that RAS can improve walking speed, cadence, symmetry, and stride length after stroke [18, 53–56], most interventions have been short-term and therapist-supervised in clinical settings, limiting applicability to daily life. MR-001’s autonomous closed-loop, sensor-driven design enables individualized gait cueing and progression without clinician oversight, offering a model that could be scalable, sustainable, and accessible beyond the clinic. This study extends the evidence base by testing MR-001 under conditions that reflect how recovery unfolds at home, in the intended use environment, where ongoing rehabilitation is often needed but rarely available.

A central aspect of this trial is its focus on engagement as the primary endpoint. Long-term adherence to rehabilitation programs is a well-recognized challenge [57, 58], particularly when delivered remotely. By tracking usage patterns, progression, and dropout patterns over extended periods, the trial will generate actionable insights into the acceptability, engagement patterns, and adherence drivers for home-based RAS. These data are critical for determining how digital therapeutics can maintain patient participation over the long term.

The trial’s withdrawal and randomized re-treatment design offers another unique contribution. This approach allows assessment of both the durability of MR-001’s effects after treatment cessation and the potential for renewed gains when the intervention is reintroduced. Such data reflect real-world usage scenarios, where access to digital therapeutics may be intermittent due to changes in coverage, functional status, or personal preference. The findings will help inform strategies for intermittent or repeated use within pragmatic care models.

Beyond clinical outcomes, this study will conduct exploratory analyses of the economic impact of home-based RAS with MR-001. Stroke-related disability is a major driver of health system burden, particularly through increased risk of falls, emergency visits, and long-term care needs. Gait speed has been described as the “sixth vital sign” because of its strong predictive value for morbidity, mortality, and healthcare utilization [59, 60]. Improvements in gait performance, like speed and quality, are strongly associated with reduced fall risk [61–63], and falls represent a leading cost driver in post-stroke care. Given the established link between walking performance and healthcare outcomes [64, 65], these exploratory analyses may provide early insights into whether gains achieved with MR-001 correspond to reduced falls, emergency visits, and long-term care burden, consistent with prior budget impact modeling that projected substantial cost savings [38]. Demonstrating such reductions could provide an evidence-based rationale for payer reimbursement and integration of digital neurorehabilitation into value-based stroke care.

Several limitations must be acknowledged. First, the open-label design of the re-treatment phase could introduce bias; however, the use of objective, performance-based outcomes (e.g., 6MWT, TUG) helps mitigate this risk [66]. Second, the study sample is limited to English-speaking participants with a minimum gait speed and a reciprocal gait pattern, which may limit generalizability to linguistically diverse and more impaired individuals. Third, recruitment requires participants to live within practical proximity of designated assessment centers and to be willing to travel for in-person visits. This geographic constraint may exclude otherwise eligible individuals and limit the applicability of findings to those with sufficient resources, mobility, and support to attend these visits. Fourth, while the 12-week intervention is longer than in many prior RAS trials, the optimal duration for sustained benefit remains unknown, and follow-up is limited to 12 months. Finally, although the trial is designed to mirror real-world delivery of MR-001, it remains a protocol-driven clinical study that provides structured support and compensates participants for their time and effort, which may influence engagement patterns and outcomes compared to routine clinical practice.

Despite these limitations, this study represents an important step toward translating validated neurorehabilitation techniques into large-scale, accessible, home-based delivery models. By integrating engagement, clinical efficacy, durability, and economic outcomes within a single study, it is positioned to deliver a comprehensive evidence base that can guide clinical guidelines, reimbursement policy, and long-term stroke care strategies. If successful, the findings will support the use of MR-001 and similar autonomous systems as scalable solutions to extend effective rehabilitation into the home and sustain recovery well into the chronic phase of stroke.

## Trial status

Protocol version 4, dated 21 September 2025. Recruitment began in October 2023 and was completed in August 2024. Although recruitment for the study has been completed, final data collection and data lock is not expected until October 2025. The protocol was not submitted earlier due to evolving operational considerations and the decision to allow the study procedures to stabilize before finalizing the manuscript.

## Data Availability

The datasets generated and analyzed during the current study will be available from the corresponding author on reasonable request, following publication of the primary results and approval of a data sharing proposal. Data sharing will be conducted in accordance with participant consent provisions and applicable regulations.

## Abbreviations

6MWT: 6-Minute Walk Test
AE: Adverse event
AESI: Adverse event of special interest
AIC: Akaike Information Criterion
AME: Auditory-motor entrainment
ATT: Average treatment effect on the treated
BIC: Bayesian Information Criterion
BI: Barthel Index
CRO: Contract Research Organization
CRIO: Clinical Research IO
CTMS: Clinical Trial Management System
DSMP: Data and Safety Monitoring Plan
ECA: External control arm
FAQ: Frequently asked questions
FDA: Food and Drug Administration
GCP: Good Clinical Practice
HEAP: Health Economics Analysis Plan
HRU: Healthcare resource utilization
IFU: Instruction for Use
IRB: Institutional Review Board
ITT: Intention-to-treat
LMM: Linear mixed model
MAR: Missing at random
MCID: Minimal clinically important difference
MR-001: MedRhythms digital therapeutic device
PHQ-8: Patient Health Questionnaire (8-item version)
PI: Principal investigator
PP: Per-protocol
PROMIS: Patient-Reported Outcomes Measurement Information System
RAS: Rhythmic auditory stimulation
RCT: Randomized controlled trial
SAE: Serious adverse event
SAP: Statistical Analysis Plan
SDV: Source data verification
Sub-I: Sub-investigator
TMT: Trail Making Test
TUG: Timed Up and Go
